# Changes in serum uteroglobin level in type 2 diabetes mellitus patients

**DOI:** 10.3389/fendo.2024.1416326

**Published:** 2024-09-30

**Authors:** Joung Youl Lim, Sang-Hyeon Ju, Ji Min Kim, Hyon-Seung Yi, Ju Hee Lee, Hyun Jin Kim, Bon Jeong Ku, Kyong Hye Joung

**Affiliations:** ^1^ Department of Endocrinology and Metabolism, Chungnam National University Hospital, Daejeon, Republic of Korea; ^2^ Graduate School of Medical Science and Engineering, Korea Advanced Institute of Science and Technology, Daejeon, Republic of Korea; ^3^ Department of Endocrinology and Metabolism, Chungnam National University Sejong Hospital, Sejong, Republic of Korea; ^4^ Department of Internal Medicine, Chungnam National University College of Medicine, Daejeon, Republic of Korea

**Keywords:** uteroglobin, SCGB1A1, prediabetes, type 2 diabetes mellitus, dyslipidaemia, metformin, statin

## Abstract

**Background:**

Uteroglobin is a multifunctional protein with anti-inflammatory properties. Studies have revealed the importance of inflammation in type 2 diabetes mellitus (T2D) pathogenesis. Here, we investigated the relationship between uteroglobin and T2D.

**Methods:**

We performed diagnostic tests for diabetes in subjects who had not been diagnosed with or treated for T2D. We established three groups, containing those with normal glucose tolerance (NGT), prediabetes and T2D, consisting of 80 people each, and compared their uteroglobin levels. In addition, 28 patients newly diagnosed with T2D were treated with metformin for 12 weeks, and 63 patients newly diagnosed with dyslipidaemia during the treatment for T2D were treated with statin for 12 weeks.

**Results:**

This study showed that uteroglobin levels were significantly lower in prediabetes and T2D groups than in the NGT group. Uteroglobin levels were not significantly correlated with other metabolic parameters, except BMI, HOMA-β and eGFR. In the group treated with metformin or statin, uteroglobin levels increased after treatment compared to before treatment.

**Conclusions:**

Uteroglobin is a sensitive factor that was decreased even in prediabetes and increased upon treatment with drugs with anti-inflammatory effects. Uteroglobin is a potential early biomarker that reflects a chronic inflammatory condition in T2D.

## 1 Introduction

Uteroglobin (Secretoglobin family 1a member 1, SCGB1A1) is a small protein (10 kDa) discovered in the uterus of rabbits in 1967 (1), and is known to be present only in mammals (2). It is included within the secretoglobin protein family, which consists of small, secreted proteins exhibiting diverse physiological roles (2). Uteroglobin exerts important anti-inflammatory functions through various mechanisms including the inhibition of phospholipase A2, which produces multiple lipid mediators, such as leukotrienes that promote inflammation and modulate immune responses (3, 4). It has been reported that uteroglobin deficiency in animals causes excessive inflammation and, in humans, uteroglobin is reduced in chronic inflammatory lung diseases such as asthma and chronic obstructive pulmonary disease (5).

The importance of inflammation in type 2 diabetes mellitus (T2D) pathogenesis has been identified. Various biological pathways related to inflammation contribute to diabetes, and exacerbated hyperglycaemia also promotes inflammation (6). For example, a high glucose level itself causes oxidative stress through activation of the polyol pathway, formation of advanced glycation products (AGEs) and other mechanisms, which not only directly causes beta-cell dysfunction but also induces inflammatory cytokine production (7). Obesity is also a major contributor to inflammation, with the accumulation of adipose tissue macrophages in excess adipose tissue, leading to the activation of inflammatory pathways (8). Cytokines and other inflammatory mediators produced as a consequence of inflammation interfere with insulin signalling and lead to insulin resistance and pancreatic beta-cell dysfunction (9). Therefore, to prevent and treat T2D, it is important to manage inflammation, and several anti-inflammatory agents have been shown to reduce insulin resistance and improve glucose control (10).

The drug metformin, which is most commonly used in T2D, is reported to have anti-inflammatory functions as well as a blood glucose-lowering effect (11). Statins, which are used for dyslipidaemia, a common condition in T2D, are also known to have anti-inflammatory effects (12). Although these mechanisms behind these anti-inflammatory activities remain incompletely understood and are still being studied, it has been reported that both metformin and statins can reduce the levels of inflammatory markers such as tumour necrosis factor-α (TNF-α), interleukin-6 (IL-6) and high-sensitivity C-reactive protein (hsCRP) (11, 13). In addition to laboratory test results, the impact of metformin and statins on inflammatory diseases can also be observed in epidemiological and clinical studies. Among diabetes patients, those treated with metformin have been found to have a lower risk of developing COPD (14), and be a slowed the decline of lung function in those with COPD (15). It has been observed that the incidence of inflammatory bowel disease (16) and the risks associated with colonic diverticula, including acute diverticulitis, are reduced among metformin users (17). Additionally, statins have been found to slow the rate of decline in lung function and improve exercise capacity in patients with COPD (18, 19). In asthma, a double-blind randomised controlled trial showed that short-term administration of atorvastatin improved subjective symptoms (20). It has been observed that patients exposed to statins have a reduced incidence of inflammatory bowel disease (21).

As far as we are aware, how the anti-inflammatory uteroglobin is associated with T2D, a condition in which inflammation is important, has not been investigated. As such, this study was implemented with the goals of identifying the relationship between serum uteroglobin level and T2D, and also determining how uteroglobin level changes after metformin or statin treatment.

## 2 Materials and methods

### 2.1 Study design

This work was planned in the form of a retrospective study using blood serum samples kept at the Human Resource Bank of Chungnam National University Hospital (CNUH; tertiary referral hospital in Daejeon, South Korea). The banked sets analysed were all samples stored for the purpose of discovering new biomarkers related to the diagnosis, treatment, prognosis, and complications of T2D. To draw comparative results among groups, criteria for each group were established, and patients who voluntarily visited the Department of Endocrinology and Metabolism (DEM) of CNUH, without any prior knowledge of the research and met the criteria were informed about the cohort construction and donated their blood and urine samples to the hospital. The entirety of the experimental protocol followed the tenets of the Declaration of Helsinki and its later amendments, while approval from the institutional review board (IRB), the Ethics Committee of CNUH, was also obtained. All participants were informed of the study’s purpose and gave their consent in written form. The detailed inclusion and exclusion criteria for each banked set are described in Supplementary Inclusion and Exclusion Criteria in **Supplementary Material**. Among the various potential candidates related to T2D we thought, uteroglobin in serum was measured in 2019 in three sets of banked samples, as described below. To minimise confounding variables, there were no changes to other medications during the study period. To minimise selection bias, all stored samples were measured and analysed for uteroglobin without exception.

The first banked set (IRB No. 2014-12-013, approval date: 6 Feb, 2015) consisted of samples of 240 individuals subjected in an outpatient setting at the DEM of CNUH to a glucose tolerance test (75 g, glucose administered orally: OGTT) from January 2014 to December 2016. These subjects were divided into three groups according to the test results: normal glucose tolerance (NGT), prediabetes and T2D (n=80 in each group). The diagnoses of prediabetes and T2D were based on the American Diabetes Association diagnostic criteria: fasting plasma glucose (FPG), 2-h plasma glucose after the 75 g OGTT (post-load 2-h PG) and HbA1_C_.

The second banked set (IRB No. 2014-12-013, approval date: 6 Feb, 2015) consisted of samples of 28 people diagnosed with diabetes who commenced treatment for it for the first time from January 2014 to December 2016. These individuals were treated with 500 mg of an extended-release formulation of metformin once daily on an outpatient basis at the DEM of CNUH. In the second banked set, serum uteroglobin was measured in samples at the start of metformin treatment and after 12 weeks of treatment to compare uteroglobin levels between before and after metformin treatment.

The third banked set (IRB No. PMS2017-005, approval date: 19 Apr, 2017) consisted of samples of 63 patients treated for diabetes and commencing statin treatment for dyslipidaemia from October 2017 to March 2019. All subjects had been allocated in a randomised and double-blinded manner at a 1:1 ratio to a group given a 5 mg dose of rosuvastatin or a group given doses of 5 mg of rosuvastatin along with 10 mg of ezetimibe on an outpatient basis at the DEM of CNUH. The third banked set was used to compare uteroglobin levels between before and 12 weeks after statin treatment.

### 2.2 Clinical parameters

Based on the American Diabetes Association’s Standards of Care in Diabetes guidelines, specifically the Comprehensive Medical Evaluation and Assessment of Comorbidities (22), we conducted medical history taking, physical examinations and laboratory examination in the same manner as we routinely do when managing diabetes patients in our hospital. For all study participants, baseline data of age, sex, smoking history, and current use of medications for hypertension and dyslipidaemia, as well as results of physical examinations including of body weight, height, and systolic and diastolic blood pressure (BP) on the day of the blood test, had been recorded. To measure BP, an automatic blood pressure monitor was used on the right arm of each participant, who had been placed in a seated position and allowed to rest for 20 min. BMI was recorded in the unit of kg/m^2^.

### 2.3 Biochemical parameters

Venous blood collection and the OGTT with 75 g of glucose were performed in the morning after an overnight fast for at least 8 h. Serum was separated after coagulation by centrifugation for 15 min at 1,000 g. Some blood was immediately transferred to the Human Resource Bank of CNUH for storage, where serum was separated and subsequently stored in aliquots at –80°C. Fasting blood samples were used to measure the levels of blood urea nitrogen (BUN), triglycerides (TG), creatinine (Cr), glucose, insulin, C-peptide, hsCRP, low-density-lipoprotein (LDL) cholesterol, high-density-lipoprotein (HDL) cholesterol and total cholesterol. We also measured the levels of glucose, insulin and C-peptide 2 h after the OGTT. An automated blood chemistry analyser (TBA-FX8, Canon) was employed to measure all of these variables. High-performance liquid chromatography was applied in line with the National Glycohemoglobin Standardization Program to determine the level of HbA1_C_, with standardisation of the results to the assay reference of the Diabetes Control and Complications Trial (23). Moreover, a commercially available human enzyme-linked immunosorbent assay (ELISA) kit was used to determine the levels of uteroglobin in serum (R&D Systems, Inc., Minneapolis, MN, USA), following the manual of the manufacturer. In our laboratory, the ELISA kit results were determined to have an intra- and interassay coefficients of variation (CV) of 6.4% and 10.5%, respectively.

### 2.4 Calculation of biochemical parameters

For calculation of the homeostasis model assessment of insulin resistance (HOMA-IR), the following formula was used: [fasting glucose level (mg/dL) × fasting insulin level (µIU/mL)]/405. Meanwhile, the following was applied for the homeostasis model assessment of beta-cell index (HOMA-β): 360 × fasting insulin level (µIU/mL)/]fasting glucose level (mg/dL) – 63] (24). The estimated glomerular filtration rate (eGFR) was obtained using a modified version of the diet in renal disease (MDRD) equation of the National Kidney Foundation (NKF) (25).

### 2.5 Statistical analysis

In this paper, data are presented as mean ± standard deviation (SD) for continuous variables, but as count (percentage) for categorical ones. Here, one-way analysis of variance (ANOVA) was employed for continuous variable-based comparisons between groups, while chi-squared test was used for categorical variables, with Bonferroni’s significant difference *post hoc* test. Meanwhile, adjustment for covariates was performed using analysis of covariance (ANCOVA). Additionally, Pearson’s correlation analysis along with multiple linear regression was employed here to evaluate the relationships between the parameters. Meanwhile, the changes in parameters between before and after treatment were compared among the groups using a paired sample t-test. For nonparametric variables, the Wilcoxon test was used. As a threshold for significance, statistical significance was defined at < 0.05 as a two-tailed *p-*value. Statistical analysis was performed using SPSS version 26.0 software (IBM, Chicago, IL, USA).

## 3 Results

### 3.1 Relationship between serum uteroglobin levels and glycaemic status

#### 3.1.1 Clinical and laboratory characteristics

**Figure 1:**
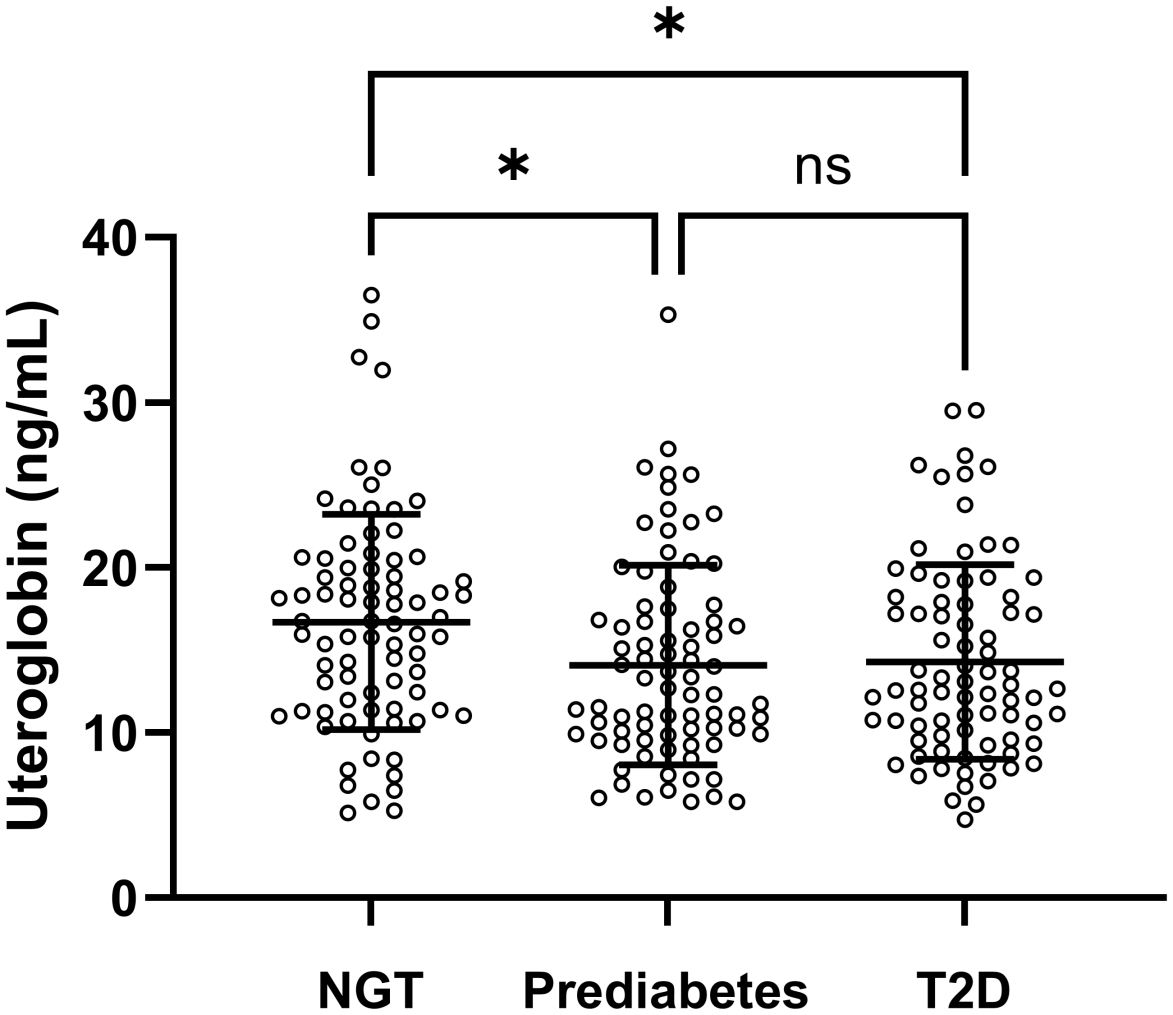
Serum uteroglobin levels among the NGT, prediabetes and T2D groups in the first banked set. Lines indicate mean and SD. NGT, normal glucose tolerance; T2D, type 2 diabetes mellitus; ns, not significant. *p*-values were calculated by one-way ANOVA. Differences at *p* < 0.05 determined by Bonferroni’s significant difference *post hoc* test are expressed as an asterisk (*).

Clinical and laboratory characteristics of the members of the NGT, prediabetes and T2D groups in the first banked set are summarised in **Table 1**. In this study comparing the NGT, prediabetes and T2D groups, the mean age of the 240 participants was 52.7 years (range 18–82 years old). The prediabetes group had higher levels of post-load 2-h PG and insulin, and a lower level of HDL cholesterol than the NGT group. Compared with the NGT group, the T2D group had higher rates of smoking, dyslipidaemia and hypertension. The T2D group also showed higher levels of weight, BMI, HbA1C, fasting plasma glucose (FPG), post-load 2-h PG, fasting insulin, post-load 2-h insulin, fasting C-peptide, HOMA-IR and TG, along with lower HDL cholesterol levels. Moreover, compared with the prediabetes group, the T2D group tended to show elevated levels of HbA1_C_, FPG, post-load 2-h PG, fasting C-peptide and HOMA-IR. A significant difference (*p =* 0.013) *in utero*globin levels (ng/mL) was also identified among the three groups (**Figure 1** and **Supplementary Table 1**). In the *post hoc* analysis, uteroglobin levels were also shown to differ significantly between the NGT and prediabetes groups (16.7 ± 6.5 vs. 14.1 ± 6.0, *p =* 0.022) and between the NGT and T2D groups (16.7 ± 6.5 vs. 14.3 ± 5.9, *p =* 0.037), while the prediabetes and T2D groups did not differ significantly (14.1 ± 6.0 vs. 14.3 ± 5.9, *p* = 0.989) in this regard. Even after adjusting for smoking status, presence of dyslipidaemia and hypertension, a statistically significant difference *in utero*globin levels among the three groups remained (*p* = 0.012) (**Supplementary Table 1**).

**Table 1:** Comparison of clinical and laboratory characteristics between participants with NGT, prediabetes and T2D.

	NGT (n=80)	Prediabetes (n=80)	T2D (n=80)	*p-*value
Age (years)	50.7 ± 13.7	53.5 ± 12.1	54.0 ± 12.3	0.210
Male (%)	25 (31.3)	38 (47.5)	35 (43.8)	0.091
Smoking (%)	8 (10.0)	7 (8.8)	22 (27.5) ^‡^	0.001
On dyslipidaemia medication (%)	9 (11.3)	12 (15.0)	22 (27.5) ^‡^	0.019
On hypertension medication (%)	16 (20.0)	14 (17.5)	33 (41.3) ^‡^	0.001
Height (cm)	161.6 ± 8.6	163.2 ± 8.4	163.3 ± 9.4	0.528
Weight (kg)	63.9 ± 12.4	67.1 ± 11.9	70.1 ± 14.7 *	0.039
BMI (kg/m2)	24.3 ± 3.3	24.8 ± 3.2	26.2 ± 4.4 *	0.020
SBP (mmHg)	127.8 ± 16.1	128.9 ± 14.6	134.6 ± 20.2	0.065
DBP (mmHg)	79.3 ± 12.6	77.4 ± 10.9	81.8 ± 11.8	0.102
HbA1_C_ (%)	5.3 ± 0.2	5.6 ± 0.3	7.4 ± 1.9 *^§^	< 0.001
Fasting PG (mg/dL)	91.9 ± 5.0	101.7 ± 11.1	154.2 ± 57.3 *^§^	< 0.001
Post-load 2-h PG (mg/dL)	105.3 ± 19.0	143.3 ± 33.5 *	293.0 ± 101.2 *^§^	< 0.001
Fasting insulin (μU/mL)	8.3 ± 4.2	10.0 ± 5.0	18.0 ± 38.9 *	0.019
Post-load 2-h insulin (μU/mL)	36.6 ± 43.6	68.9 ± 68.0 *	70.7 ± 69.5 *	0.001
Fasting c-peptide (ng/dL)	0.73 ± 0.47	0.87 ± 0.50	1.15 ± 0.71 *^§^	< 0.001
Post-load 2-h c-peptide (ng/dL)	3.4 ± 2.2	4.1 ± 2.7	6.7 ± 24.3	0.347
HOMA-IR	1.9 ± 1.0	2.5 ± 1.3	7.2 ± 19.1 *^§^	0.005
HOMA-β	103.9 ± 52.0	104.5 ± 76.5	84.9 ± 111.4	0.249
Total cholesterol (mg/dL)	191.4 ± 30.4	196.6 ± 39.8	192.3 ± 46.9	0.628
TG (mg/dL)	104.0 ± 63.2	145.1 ± 106.7	189.6 ± 173.0 *	< 0.001
HDL cholesterol (mg/dL)	61.8 ± 13.7	53.7 ± 14.0 *	49.0 ± 13.4 *	< 0.001
LDL cholesterol (mg/dL)	113.5 ± 29.7	121.5 ± 34.2	116.6 ± 45.3	0.389
eGFR (mL/min/1.73m2)	103.4 ± 18.1	104.2 ± 24.4	112.0 ± 29.4	0.061
hsCRP (mg/L)	1.0 ± 2.6	1.5 ± 1.8	2.7 ± 5.5	0.078

The data are expressed as the mean ± SD or number (%). NGT, normal glucose tolerance; T2D, type 2 diabetes mellitus; BMI, body mass index; SBP, systolic blood pressure; DBP, diastolic blood pressure; PG, plasma glucose; HOMA-IR, homeostasis model assessment for insulin resistance; HOMA-β, homeostasis model assessment for beta-cell function; TG, triglyceride; HDL, high-density lipoprotein; LDL, low-density lipoprotein; eGFR, estimated glomerular filtration rate; hsCRP, high-sensitivity C-reactive protein. p-values were calculated by one-way ANOVA or chi-squared test according to Bonferroni’s significant difference post hoc test. Differences at p < 0.05 are expressed as follows: *, NGT vs. prediabetes and NGT vs. T2D; ^§^, prediabetes vs. T2D; ^‡^, p < 0.05 for chi-squared test.

#### 3.1.2 Relationship between serum uteroglobin levels and various parameters

The associations between serum uteroglobin levels and various metabolic parameters were also examined in this work. Pearson’s correlation analysis was used to determine the correlations with various metabolic parameters, as shown in **Table 2**. BMI, HOMA-β and eGFR were negatively correlated with uteroglobin levels (BMI: *r* = −0.180, *p* = 0.017, HOMA-β: *r* = −0.134, *p* = 0.039, eGFR: *r* = −0.247, *p* < 0.001). Meanwhile, no strong correlations with uteroglobin levels were found for any of the metabolic parameters that we investigated.

**Table 2:** Correlations between serum uteroglobin level and various parameters.

	*r*	*p-*value
Age (years)	0.118	0.068
BMI (kg/m2)	-0.180	0.017
SBP (mmHg)	-0.042	0.576
DBP (mmHg)	-0.058	0.443
HbA1_C_ (%)	0.012	0.857
Fasting PG (mg/dL)	-0.032	0.627
Post-load 2-h PG (mg/dL)	-0.028	0.667
Fasting insulin (μU/mL)	-0.104	0.110
Post-load 2-h insulin (μU/mL)	-0.106	0.115
Fasting c-peptide (ng/dL)	-0.124	0.068
Post-load 2-h c-peptide (ng/dL)	-0.033	0.628
HOMA-IR	-0.087	0.182
HOMA-β	-0.134	0.039
Total cholesterol (mg/dL)	0.013	0.841
TG (mg/dL)	0.001	0.993
HDL cholesterol (mg/dL)	-0.006	0.931
LDL cholesterol (mg/dL)	0.037	0.581
eGFR (mL/min/1.73m^2^)	-0.247	0.001
hsCRP (mg/L)	-0.104	0.198

Data are expressed as Pearson’s correlation coefficients. BMI, body mass index; SBP, systolic blood pressure; DBP, diastolic blood pressure; PG, plasma glucose; HOMA-IR, homeostasis model assessment for insulin resistance; HOMA-β, homeostasis model assessment for beta-cell function; TG, triglycerides; HDL, high-density lipoprotein; LDL, low-density lipoprotein; eGFR, estimated glomerular filtration rate; hsCRP, high-sensitivity C-reactive protein.

### 3.2 Changes in serum uteroglobin levels with drug treatment

#### 3.2.1 Changes in serum uteroglobin levels with metformin treatment

**Figure 2:**
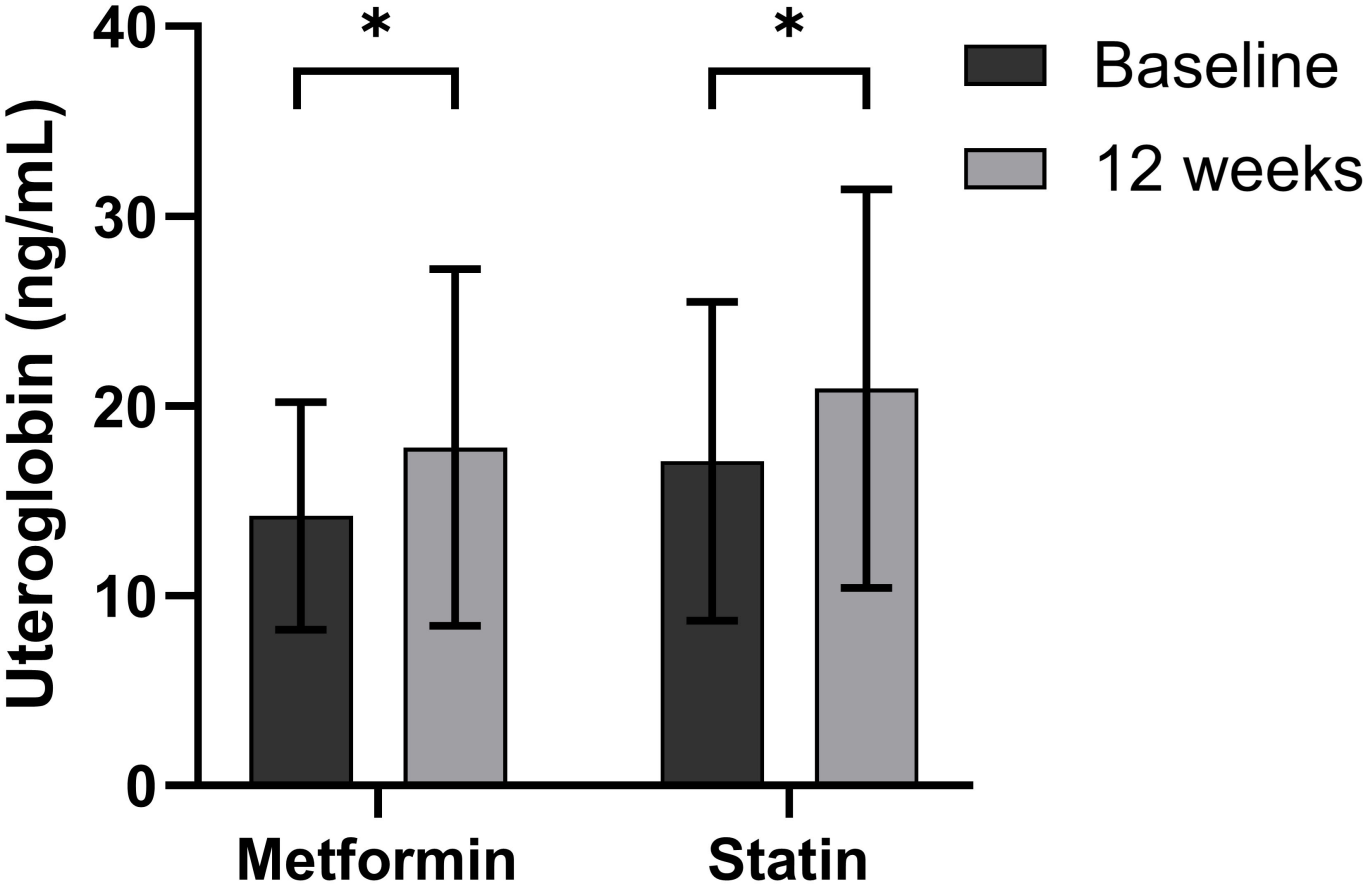
Changes in serum uteroglobin level after 12 weeks of metformin or statin in type 2 diabetes mellitus patients. Boxes and lines indicate mean and SD. Differences at *p* < 0.05 determined by paired *t* test are expressed as an asterisk (*).

The participants in the second banked set were found to have a mean age of 51.5 years (range 20–77) (**Supplementary Table 2**). **Table 3** and **Figure 2** show the changes *in utero*globin and other parameters after 12 weeks of metformin treatment. Uteroglobin level was significantly increased (from 14.2 ± 6.0 to 17.8 ± 9.4, *p* = 0.012) while HbA1_C_, total and LDL cholesterol, and eGFR were significantly decreased by metformin. Meanwhile, weight, BMI, TG and HDL cholesterol showed no significant changes.

**Table 3:** Changes in serum uteroglobin level and various parameters after 12 weeks of metformin treatment.

	Baseline	After treatment	*p-*value
Uteroglobin (ng/mL)	14.2 ± 6.0	17.8 ± 9.4	0.012*
Weight (kg)	66.8 ± 13.3	66.8 ± 13.4	0.725
BMI (kg/m2)	24.9 ± 3.0	24.7 ± 3.1	0.827
HbA1_C_ (%)	8.4 ± 1.6	6.8 ± 0.8	< 0.001
Total cholesterol (mg/dL)	188.4 ± 38.9	158.1 ± 37.3	0.003
TG (mg/dL)	169.4 ± 113.6	154.6 ± 95.1	0.554
HDL cholesterol (mg/dL)	46.8 ± 10.5	47.0 ± 10.9	0.807
LDL cholesterol (mg/dL)	119.7 ± 38.5	91.7 ± 29.9	0.002
eGFR (mL/min/1.73m^2^)	114.8 ± 28.9	107.1 ± 26.8	0.009

The data are expressed as the mean ± SD. p-values were calculated by paired t test for parametric variables and Wilcoxon test for nonparametric variables (*). BMI, body mean index; TG, triglyceride; HDL, high-density-lipoprotein; LDL, low-density-lipoprotein; eGFR, estimated glomerular filtration rate.

#### 3.2.2 Changes in serum uteroglobin levels with statin treatment

The participants in the third banked set were found to have a mean age of 57.6 years (range 28–83) (**Supplementary Table 3**). **Table 4** and **Figure 2** show the changes *in utero*globin and other parameters after 12 weeks of statin treatment. Uteroglobin level was significantly increased (from 17.1 ± 8.4 to 20.0 ± 10.5, *p* < 0.001) and HDL cholesterol was also increased, while fasting insulin, HOMA-β, total and LDL cholesterol, and TG were significantly decreased after statin treatment.

**Table 4:** Changes in uteroglobin level and various parameters after 12 weeks of statin treatment.

	Baseline	After treatment	*p*-value
Uteroglobin (ng/mL)	17.1 ± 8.4	20.9 ± 10.5	< 0.001
Weight (kg)	69.8 ± 12.7	69.6 ± 12.9	0.514
BMI (kg/m2)	26.5 ± 4.1	26.4 ± 4.1	0.367
HbA1_C_ (%)	6.9 ± 1.1	6.9 ± 0.9	0.780
Fasting PG (mg/dL)	151.1 ± 51.2	144.9 ± 39.5	0.382
Fasting Insulin (μIU/mL)	17.2 ± 10.6	13.2 ± 10.8	0.010
HOMA-IR	6.5 ± 4.6	5.2 ± 6.7	0.148
HOMA-β	87.8 ± 67.7	65.2 ± 39.4	0.003
Total cholesterol (mg/dL)	213.6 ± 30.2	151.0 ± 34.0	< 0.001
TG (mg/dL)	195.3 ± 106.5	149.62 ± 74.5	< 0.001
HDL cholesterol (mg/dL)	47.0 ± 12.0	49.3 ± 12.7	0.012
LDL cholesterol (mg/dL)	137.3 ± 23.6	81.4 ± 29.3	< 0.001
eGFR (mL/min/1.73m2)	107.1 ± 23.3	105.8 ± 22.6	0.408

The data are expressed as the mean ± SD or number (%). BMI, body mass index; PG, plasma glucose; HOMA-IR, homeostasis model assessment for insulin resistance; HOMA-β, homeostasis model assessment for beta-cell function; TG, triglycerides; HDL, high-density lipoprotein; LDL, low-density lipoprotein; eGFR, estimated glomerular filtration rate. p-values were calculated by paired t test.

## 4 Discussion

This is the first study to show a meaningful correlation between serum uteroglobin levels and T2D in humans. To identify the association between diabetes and uteroglobin, we used three banked sets from the Human Resource Bank. The first banked set was based on subjects with a status of NGT or diagnosed with prediabetes or T2D for the first time. The second and third banked sets were based on first-time users of metformin or statin, among patients with diabetes. We compared the three groups, namely, NGT, prediabetes and T2D, and found that uteroglobin differed significantly among them. Uteroglobin decreased in the prediabetes and T2D groups compared with that in the NGT group. In patients with T2D, the administration of metformin or statin recovered the uteroglobin level. Higher BMI was associated with lower uteroglobin. As confirmed in other studies, higher eGFR was associated with a decrease in serum uteroglobin (26).

Uteroglobin is a protein that itself has anti-inflammatory function, primarily by inhibiting phospholipase A2, which reduce leukotriene synthesis (3). In humans, uteroglobin has also been linked to certain health conditions (27). For example, it has been associated with respiratory diseases such as asthma, allergic rhinitis and idiopathic pulmonary fibrosis, and kidney diseases such as IgA nephropathy (27, 28). These diseases are associated with chronic inflammation, and asthma sufferers have reduced levels of uteroglobin, while polymorphisms of uteroglobin can affect the rate of progression of IgA nephropathy (27, 28). Recombinant uteroglobin has also been shown to improve renal disease in animal studies (29) and lung disease associated with prematurity in clinical studies (30, 31). T2D is also associated with chronic inflammation (6), and this study found a unique relationship between uteroglobin and T2D. When comparing the NGT, prediabetes and T2D groups, they were shown to differ significantly *in utero*globin levels. Instead of a gradual change with the degree of hyperglycaemia, there was no difference between the prediabetes and T2D groups, and interestingly only the differences between NGT and prediabetes groups and between NGT and T2D groups were found to be significant. Meanwhile, uteroglobin was shown to be weakly negatively correlated with HOMA-β. Moreover, it was not found to be strongly correlated with other metabolic parameters or even with hsCRP, an inflammatory biomarker for which associations with vascular complications in diabetic patients have been reported (32).

Managing diabetes requires a diverse approach that addresses various pathophysiological aspects of the disease, not just glucose control. One of the key axes in the pathophysiology of T2D is inflammation. Due to the various aetiologies, even with well-controlled blood glucose levels, and even in prediabetes, the risk of vascular complications is significantly higher compared to NGT (33). Diabetes is classified as a high-risk group for various vascular diseases, and it is recommended that LDL levels be maintained lower than in non-diabetic cases (34). Intensive glucose control has not been able to drastically reduce macrovascular complications, thus it is crucial to continue efforts to identify new factors related to the progression and complications of T2D and prediabetes (35). This study’s identification of a relationship between uteroglobin, a protein with inherent anti-inflammatory properties, and T2D, where low-grade chronic inflammation is a key pathophysiological aspect, is highly significant. The lack of strong correlations with existing metabolic parameters suggests that uteroglobin might act as an independent, critical new axis, indicating the need for large-scale studies to explore its relationship with diabetes complications. Furthermore, even aside from diabetes, understanding what interventions (in this study, metformin and rosuvastatin) change uteroglobin levels and how these changes subsequently affect the body remains an unexplored field.

One of the most important and common complications related to T2D is atherosclerosis (36). Atherosclerosis is not just an accumulation of cholesterol, inflammation also plays a crucial role (37). Many experimental studies, including on immune-mediated atherosclerosis after allogeneic transplantation, have shown that inflammation-related cells such as macrophages and many cytokines are also important in atherosclerosis (38). Preclinical studies have also shown that controlling inflammation with various anti-inflammatory drugs can reduce the severity of atherosclerosis (39). Metformin and statins have anti-inflammatory benefits beyond lowering blood sugar and lowering LDL cholesterol, respectively (11, 12). Metformin is still widely used as a first-line treatment worldwide, except in cases of heart failure, chronic kidney disease or myocardial infarction (40). The anti-inflammatory effects as well as glucose-lowering effects of metformin have been a focus of numerous preclinical and clinical studies. These studies have found that the effects of metformin on the inflammatory response range from the organ level, such as adipose tissue, heart and blood vessels, to the cellular level, including T cells, macrophages and B cells (11). Findings have implied that such effects are mediated primarily by AMP kinase activation along with downstream effects of the inhibition of mTOR and NF-κB pro-inflammatory signalling cascades (11). These anti-inflammatory effects mean that, in patients with T2D, metformin does more than just lower blood glucose, especially in the context of atherosclerosis (41). Statins are most commonly used to lower LDL cholesterol, which is one of the most important causes of atherosclerosis (42). Statins are known to stabilise atheroma by not only lowering LDL cholesterol, but also by reducing inflammation (37). A major part of the anti-inflammatory function of statins is mediated by the inhibition of isoprenoid, which affects various signalling systems, especially proteins that bind to guanine triphosphate (GTP) (e.g., components of the Rac, Rho and Ras pathways) (43). As a result, statins reduce inflammatory cytokines in inflammatory cells and vascular endothelial cells, reduce oxidative stress by upregulating endothelial nitric oxide synthase (eNOS) gene expression in vascular cells and modulate platelet function to inhibit thrombus formation by regulating the interaction between platelets and endothelial cells (43).

There is an interesting association between uteroglobin and vascular complications in T2D. In animal experiments, uteroglobin was found to reduce neointimal hyperplasia associated with patency after angioplasty, stenting or bypass surgery (44–46). Uteroglobin could thus help to predict the outcomes of vascular interventions. In our study, we found that metformin and statins increased uteroglobin, a protein with anti-inflammatory functions. Therefore, one of the mechanisms by which metformin and statins exert anti-inflammatory effects to prevent vascular complications may be through increasing uteroglobin.

Chronic inflammation, which is an important aetiology of diabetes, is also thought to be an important factor in the development and progression of cancers (47), and major areas of cancer research continue to focus on the relationship between the cancer and inflammatory responses (48). Interestingly, some studies on cancer suggested a potential role of uteroglobin. For example, it has been suggested that the level of uteroglobin expression in human prostate cancer tissues was negatively correlated with Gleason score (49). Preclinical experimental study suggested that treating prostate cancer cells with recombinant human uteroglobin or transfecting them with the uteroglobin gene reduced the extent of cancer cell invasion (50). In lung cancer, the level of uteroglobin expression was positively correlated with prognosis (51). Another study on lung cancer reported that the effectiveness of combination therapy with immune checkpoint inhibitors and radiotherapy was increased by induction of the uteroglobin gene expression and decreased by deletion of this gene (52). Additionally, some studies on the effects of metformin have shown potential benefits through uteroglobin in prostate cancer and prostate-related disease such as benign prostatic hyperplasia and prostatitis (53, 54). It is very well known that the incidence of various cancers increases in diabetes (55). Therefore, the discovery of uteroglobin in diabetes might open new directions in exploring the relationship between diabetes and cancer, the anti-cancer effects of drugs such as metformin and statin, and clinical implications for the prevention of cancers.

This study had some limitations. This study was conducted at a single centre and used banked samples, which were collected from individuals who sought medical care at CNUH. The sample size was also quite small. This introduces the possibility of selection bias, as the samples may not represent the general population. In diseases like T2D, which have diverse aetiologies, biomarker studies often face large inter-individual variability, and studies with small sample size frequently fail to achieve statistical significance. Although we confirmed statistically significant changes of uteroglobin in all groups of our study, larger-scale studies should be needed to suggest normal reference ranges and cutoff value for uteroglobin to predict disease.

Unfortunately, it was also not possible to obtain samples associated with metformin treatment in the prediabetic stage because of the national health insurance policy in South Korea, where it is illegal to prescribe metformin to patients with prediabetes. We also expected that ezetimibe would exert an effect in the statin-treated banked set but were unable to find an effect of ezetimibe on the level of uteroglobin. This study focused on identifying associations rather than elucidating underlying mechanisms. Future research should aim to investigate the biological pathways through which uteroglobin levels are modulated and how they influence the pathogenesis or management of T2D.

## 5 Conclusions

The present study indicates that uteroglobin is a sensitive inflammatory biomarker, the level of which can be altered even in prediabetes or upon short-term treatment with metformin and rosuvastatin, drugs with anti-inflammatory effects. More studies are required to investigate possible mechanisms by which metformin and statin influence the increase of uteroglobin and the clinical outcomes associated with these differences.

## Data Availability

The raw data supporting the conclusions of this article will be made available by the authors, without undue reservation.
