# Supplementary material for: Changes in serum uteroglobin level in type 2 diabetes mellitus patients
Source: Front Endocrinol (Lausanne). 2024 Sep 30;15:1416326. doi: 10.3389/fendo.2024.1416326 (PMC11471534; doi:10.3389/fendo.2024.1416326)
Supplement: Supplementary file 1 [file DataSheet1.docx]

Supplementary Material

Changes in serum uteroglobin level in type 2 diabetes mellitus patients

Joung Youl Lim ^1^, Sang-Hyeon Ju ^2^, Ji Min Kim ^3,4^, Hyon-Seung Yi ^1,4^, Ju Hee Lee ^1,4^, Hyun Jin Kim ^1,4^, Bon Jeong Ku ^1,4,*^, Kyong Hye Joung ^3,4,*^

*** Correspondence:** Kyong Hye Joung: [babehorse@cnuh.co.kr](mailto:babehorse@cnuh.co.kr), Bon Jeong Ku: bonjeong@cnu.ac.kr

**Supplementary inclusion and exclusion criteria 1.**

# IRB No. 2014-12-013

## Inclusion criteria

### Subgroup 1 : age ≥ 18 years, those who had not been diagnosed with T2D or were not taking oral diabetes medication or had prediabetes.

### Subgroup 2 : age ≥ 18 years, those who are starting medication for T2D for the first time and are treated with metformin extended-release formulation 500 mg once daily.

## Exclusion criteria :

- Pregnancy

- Cardiovascular disease (CVD) including ischemic heart disease, heart failure

- Stroke

- Malignant disease

- Clinical signs of acute infection or inflammation

- Advanced renal or liver disease

# IRB no. PMS2017-005

## Inclusion criteria : patients with a diagnosis of hypercholesterolemia and who have not previously treated with statins.

## Exclusion criteria

- Hypersensitivity to rosuvastatin, ezetimibe or any of its constituents

- Active liver disease or unexplained persistently elevated serum aminotransferase levels.

- Muscular diseases

- Receiving concomitant cyclosporine or fibrate

- Estimated glomerular filtration rate (eGFR) < 30 mL/min

- Pregnancy

- Hypothyroidism

- Alcoholism

- Galactose intolerance, Lapp lactase deficiency, or glucose galactose malabsorption.

**Supplementary Table 1**. Uteroglobin levels between participants with NGT, prediabetes and T2D in the first banked set

|  | NGT (n=80) | Prediabetes (n=80) | T2D (n=80) | *p*-value* | *p*-value** |
| --- | --- | --- | --- | --- | --- |
| Uteroglobin (ng/mL) | 16.7±6.5 | 14.1±6.0 | 14.3±5.9 | 0.013 | 0.012 |

The data are expressed as the mean ± SD. NGT, normal glucose tolerance; T2D, type 2 diabetes mellitus. **p-*value was calculated by one-way ANOVA. ***p*-value was calculated by ANCOVA after adjusted by smoking status, presence of dyslipidemia and hypertension.

**Supplementary Table 2**. Baseline clinical characteristics of patients treated with metformin in the second banked set (n=28)

|  | Mean ± SD  Number (%) |
| --- | --- |
| Age (years) | 51.5 ± 11.8 |
| Male (%) | 18 (64.3) |
| Smoking (%) | 6 (21.4) |
| Dyslipidemia (%) | 2 (7.1) |
| Hypertension (%) | 6 (21.4) |

The data are expressed as the mean ± SD or number(%).

**Supplementary Table 3**. Baseline clinical and laboratory characteristics of patients treated with statin in the third banked set (n=63)

|  | **Mean ± SD**  **Number (%)** |
| --- | --- |
| Age (years) | 57.6 ± 11.9 |
| Male (%) | 31 (49.2) |
| Smoking (%) | 22 (34.9) |
| Hypertension (%) | 33 (52.4) |
| Height (cm) | 162.3 ± 8.5 |
| Weight (kg) | 69.8 ± 12.7 |
| BMI (kg/m2) | 26.5 ± 4.1 |
| SBP (mmHg) | 131.4 ± 16.1 |
| DBP (mmHg) | 79.8 ± 9.9 |

The data are expressed as the mean ± SD or number(%). BMI, body mass index; SBP, systolic blood pressure; DBP, diastolic blood pressure.
